# Power comparison of different methods to detect genetic effects and gene-environment interactions

**DOI:** 10.1186/1753-6561-1-s1-s74

**Published:** 2007-12-18

**Authors:** Rémi Kazma, Marie-Hélène Dizier, Michel Guilloud-Bataille, Catherine Bonaïti-Pellié, Emmanuelle Génin

**Affiliations:** 1 Université Paris-Sud, UMR-S 535, Villejuif, 94817, France; 2 INSERM UMR-S 535, Villejuif, 94817, France

## Abstract

Identifying gene-environment (G × E) interactions has become a crucial issue in the past decades. Different methods have been proposed to test for G × E interactions in the framework of linkage or association testing. However, their respective performances have rarely been compared. Using Genetic Analysis Workshop 15 simulated data, we compared the power of four methods: one based on affected sib pairs that tests for linkage and interaction (the mean interaction test) and three methods that test for association and/or interaction: a case-control test, a case-only test, and a log-linear approach based on case-parent trios. Results show that for the particular model of interaction between tobacco use and Locus B simulated here, the mean interaction test has poor power to detect either the genetic effect or the interaction. The association studies, i.e., the log-linear-modeling approach and the case-control method, are more powerful to detect the genetic effect (power of 78% and 95%, respectively) and taking into account interaction moderately increases the power (increase of 9% and 3%, respectively). The case-only design exhibits a 95% power to detect G × E interaction but the type I error rate is increased.

## Background

Gene-environment (G × E) interactions are likely to play an important role in multifactorial diseases. The detection of G × E interaction can be of major interest in epidemiological studies to help identify subgroups of the population that are at high risk of disease and at which prevention and screening programs should be targeted. The presence of interaction can conceal environmental and/or genetic effects if not considered in the analysis [1]. On the other hand, taking it into account may either enhance or reduce the power to detect genetic susceptibility factors, depending on the parameters inherent to the model underlying disease susceptibility [2,3]. With this in mind, many statistical methods have been developed in the past decades, either to directly investigate G × E interaction or to enhance detection of genetic factors by taking into account exposure status. They can be classified according to the design followed, the kind of data used, and the hypothesis tested [1,4].

The purpose of our work is to compare the power of different methods to detect the effect of Locus B and its interaction with history of tobacco use. We used the simulated data (Problem 3) of Genetic Analysis Workshop 15 (GAW15) with knowledge of the "answers" and compared four methods to test for genetic effect and/or G × E interaction. The first method, referred to as the mean interaction test (MIT) method [5], tests linkage and G × E interaction among sib pairs. It is compared to three association testing methods: a log-linear-modeling approach [6] that uses case-parent triads and a case-control design [4], both of which test for the effect of the gene and G × E interaction, and a case-only design [7] that tests for interaction only.

## Methods

One hundred replicates were studied at the disease susceptibility Locus B that controls the effect of smoking on rheumatoid arthritis risk. In each replicate, 1500 affected sib pairs were considered for the MIT, 1500 case-parent trios for the log-linear method, 1500 cases and controls for the case-control design, and only the 1500 cases for the case-only test. We also studied smaller sample sizes (500 trios and 750 cases and controls) in order to compare the three association methods for the same number of genotyped individuals. Cases were obtained by considering the first affected case in each sib-pair and controls were the first 1500 control subjects among the 2000 available for each replicate.

Because none of the single-nucleotide polymorphisms (SNPs) close to Locus B were in linkage disequilibrium with this locus, we used genotypes of all the individuals at that locus for association tests and the exact identity-by-descent (IBD) provided in the Problem 3 "answers" for the linkage test. For the exposure status, we considered the lifetime smoking status and did not account for the indirectly increased risk through smoke effect on IgM.

The four following methods were compared.

### Mean interaction test

The MIT developed by Gauderman and Siegmund [5] is an extension of the mean sharing test [8] to account for G × E interaction. It compares the proportion of alleles shared IBD, π, which is expected to be equal to 0.5 under the null hypothesis of no linkage, across the three groups of affected sib pairs differing for the number of exposed sibs (2, 1, or 0). The following regression model is used: π_*i *_= π + β(*X*_*i*_-*X*) + ε_*i*_, where π is the intercept and β the regression coefficient for the exposure, with *X*_*i *_the covariate of exposure centered on its mean *X*. We conducted analysis using the coding scheme consisting of two variables (*X*_*EE *_and *X*_*EU*_) contrasting sib pairs with 2, 1, or 0 exposed sibs. The null hypothesis of no linkage is tested by the likelihood ratio test (LRT): T_πβ _= 2[ln{L(π = 0.5, β = 0)}-ln{L(π, β)}], which follows a 50:50 mixture distribution of two and three degrees of freedom (df) χ^2^. The alternative hypothesis corresponds to linkage with or without G × E interaction.

In its original presentation, the mean interaction test method allows accounting for G × E interaction in the search for linkage but does not test for G × E interaction. We therefore developed a LRT for G × E interaction: T_β _= 2[ln{L(π, β = 0)}-ln{L(π, β)}]. This test follows a 2 df χ^2 ^distribution.

### Log-linear-modeling approach for case-parent triads

Proposed by Umbach and Weinberg [6], this method consists of comparing the conditional genotype distribution of exposed cases, given parental genotypes, versus that of unexposed cases. Briefly, case-parent triads are divided into 20 categories based on the parental genotypes, the genotype of the case, and the exposure status of the case. The expected number of triads can be expressed according to a log-linear model [3,6]. LRT are performed to test for 1) a gene effect ignoring G × E interaction (which follows a 2 df χ^2^), 2) a gene effect accounting for G × E interaction (which follows a 4 df χ^2^), and 3) a G × E interaction (which follows a 2 df χ^2^). Fit of the data with a dominant model is also tested as the true model was dominant.

### Case-control design

Case-control designs have been widely used to compare risks of developing a disease according to their genotype and exposure status [4]. Odds-ratios (OR) associated with the exposure, the genotypes, and their interaction factors are estimated and tested for significance. Three likelihood ratio tests are performed: a 2 df χ^2 ^test for genetic effect alone, a 4 df χ^2 ^test for genetic effect accounting for G × E interaction, and 2 df χ^2 ^test of GxE interaction. Fit of the data with a dominant model is tested using a 2 df LRT.

### Case-only design

Case-only studies [4,7] test the interaction between an exposure and a genotype among case subjects only. This type of design assesses the departure from a multiplicative scale, assuming independence between both factors. To test for the interaction, a 2 df LRT of homogeneity between the genotype distribution in exposed and unexposed cases is performed.

Powers of the different tests were estimated by determining the number of replicates among the 100 replicates that were significant at a nominal 0.05 type I error rate. Type I error rates to test for G × E interaction are estimated on the seven loci (A, C-H) that are not supposed to interact with lifetime smoking status.

## Results

Table 1 gives the mean proportion of alleles shared IBD in the whole sample, and in each of the three sib-pair categories of exposure. Table 2 shows the power of the different tests. We found that MIT has almost no power to detect linkage even when accounting for G × E interaction. This could have been expected given the proportion of alleles shared IBD in the whole sample and in each of the three categories based on exposure. Indeed, these proportions are very close to the null expectation of 0.5 (Table 1).

**Table 1:** Proportion of alleles shared IBD in the sib-pairs between 1500 sib pairs over 100 replicates

	π^a^	π_UU_^b^	π_EU_^c^	π_EE_^d^
Average	0.502	0.500	0.501	0.503
SD^e^	0.008	0.018	0.018	0.013
Minimum	0.485	0.464	0.455	0.480
Maximum	0.525	0.543	0.543	0.539

^a^π is the total proportion.

^b^π_UU_, proportion in sib pairs with 0 exposed sibs.

^c^π_EU_, proportion in sib pairs with 1 exposed sibs.

^d^π_EE_, proportion in sib pairs with 2 exposed sibs.

^e^SD, standard deviation

**Table 2:** Power and estimates of interaction coefficients of the four tests over 100 replicates

	Power (%)^a^			Average interaction coefficients [95% CI]	
		
Test	G+I	G	I	I_Bb_	I_BB_
Mean interaction test	6	8	12	-	-
Log-linear-modeling^b^	87	78	53	1.33 [1.03–1.71]	1.72 [1.13–1.83]
Case-control^b^	98	95	69	1.39 [0.97–1.96]	1.88 [1.08–3.10]
Case-only^b^	-	-	95	1.39 [1.05–1.72]	1.86 [1.39–2.96]
Log-linear-modeling^c^	33	23	20	1.35 [0.79–2.15]	1.79 [0.82–3.68]
Case-control^d^	79	68	42	1.41 [0.85–2.09]	1.96 [0.99–3.37]

^a ^G+I, genetic effect accounting for interaction; G, genetic effect not accounting for interaction; I, G × E interaction.

^b ^Samples of 1500 families were used corresponding to 4500 (1500 triads), 3000 (1500 cases and 1500 controls), and 1500 genotyped individuals for the log-linear-modeling, the case-control and the case-only design, respectively.

^c ^Samples of 500 triads are considered corresponding to 1500 genotyped individuals.

^d^Samples of 750 cases and 750 controls are considered here to limit the number of genotyped individuals to 1500.

**Figure 1:**
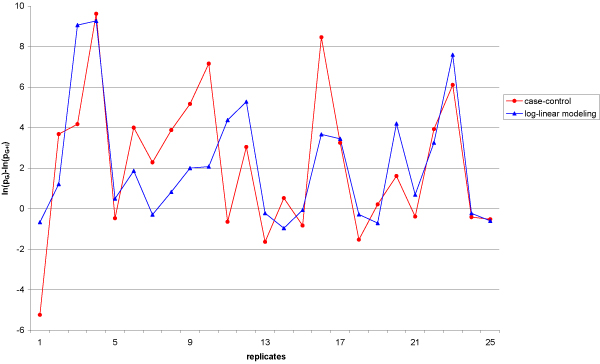
**Difference in *p*-values of G+I and G tests**. Difference is represented for the case-control (red plot) and the log-linear-modeling (blue plot) by ln(*p*_G_)-ln(*p*_G+I_) reported over the first 25 replicates.

With the log-linear model, the power to detect the gene effect is 78% and is increased to 87% when accounting for G × E interaction. Thus, there is a gain in power to detect the gene effect when accounting for G × E interaction under the simulated model. For the case-control design, the power to detect the gene effect is 95% and improves to 98% when accounting for interaction. As shown in Figure 1, the *p*-values of test accounting for G × E are smaller than those of the test not accounting for G × E for most of the replicates and similar trends (gain or loss of power) are observed between the two methods in 74% of the replicates.

**Figure 2:**
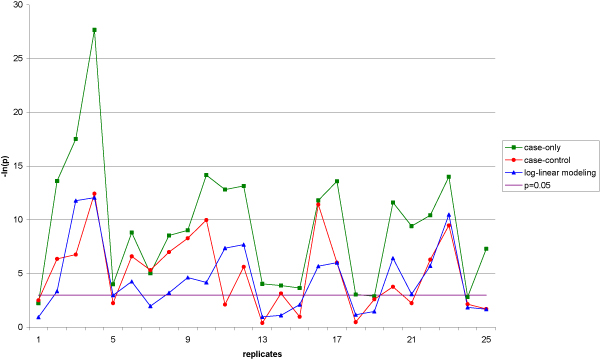
**Comparison of the *p*-values of the interaction tests**. -ln(*p*) are reported for the case-only design (green plot), the case-control design (red plot) and the log-linear-modeling method (blue plot) over the first 25 replicates.

Concerning the detection of the G × E interaction, we found that the case-only design is by far the most powerful test. It reaches 95% power; the case-control design only reaches 69%, the log-linear approach, 53%; and the linkage test (MIT), 12%. When constraining the number of genotyped individuals to be the same for the three association methods, the differences in power are even more pronounced. Figure 2 shows the *p*-values of the G × E interaction test for the log-linear-modeling approach, the case-control, and the case-only designs for the first 25 replicates. We observe that it is generally in the same replicates that the different methods give the most significant results, with the highest significance achieved for the case-only method.

Estimates of interaction factors presented in Table 2 do not seem to comply with a dominant model, and indeed a dominant model is rejected in 60% of the replicates with the case-control and in 46% of the replicates with the log-linear model.

Average type I error rates for the interaction test over the seven loci were 13% for the case-only design (ranging from 5% for Locus H to 26% for Locus C), 10% for the case-control (from 4% for Locus H to 30% for Locus C), and 8% (ranging from 3% for Loci A and F to 23% for Locus C) for the log-linear model.

## Discussion

Under the G × E simulated model presented here, it is more powerful to test for association than to test for linkage. Indeed, the MIT method has extremely poor power to detect the genetic factor either with or without taking G × E interaction into account. This could be explained by the low value of the interaction coefficient used in the simulations. Gauderman and Siegmund [5] actually showed that for an interaction coefficient less than 3 (or greater than 1/3), the MIT will not be efficient.

For the association-based approaches, accounting for the environmental factor increases the power to detect the genetic susceptibility factor from 78% to 87% for the log-linear method and from 95% to 98% for the case-control method. This gain in power is rather limited even though under the simulated model, the gene has an effect only in exposed subjects. This could be linked to the fact that the exposure is relatively frequent in the population, as shown by Selinger-Leneman et al. [3].

If one is interested in detecting the interaction, the case-only design is shown to be the most efficient. However, its validity depends on some assumptions, in particular, the independence between both genetic and environmental factors. Type I error rates were actually higher than expected (13% instead of 5%). However, it should be noted that type I errors estimated for the two other methods were also inflated. This was essentially due to Locus C, which interacts with sex and might thus indirectly be associated with tobacco exposure. When Locus C is excluded, type I error rates were close to expectation with the log-linear model (5%) and with the case-control (6%), but were still increased for the case-only design (10%).

Another point of concern was the model issue. In fact, the true model was dominant but dominance was rejected in the majority of the replicates, though less often for the log-linear method than for the case-control. A plausible explanation for this distortion could be the fact that sib pairs are ascertained, leading to a modification in expected parental genotype distributions. This is partially corrected for in the log-linear model by the conditioning on the parent genotypes.

All association approaches considered here do not take full advantage of the data because only one of the two sibs is used in each sib pair. It would be interesting to extend the methods to use the whole sibship while correcting for the dependence between the sibs.

## Conclusion

Although this study argues in favor of the use of the case-only design to detect a G × E interaction, it shows that if one is interested in detecting gene effect, accounting for the exposure is not necessary. Of course, this depends strongly on the underlying model and could probably be linked to the high exposure frequency. It will be of interest to compare the different methods presented here using a much larger range of models.

## Competing interests

The author(s) declare that they have no competing interests.
